# Chronic Type A Aortic Dissection: Rare Presentation of Incidental Pericardial Effusion

**DOI:** 10.1155/2019/3562871

**Published:** 2019-05-02

**Authors:** Ashraf Abugroun, Ahmed Subahi, Safwan Gaznabi, Hussein Daoud

**Affiliations:** ^1^Department of Internal Medicine, Advocate Illinois Masonic Medical Center, 836 W Wellington Avenue, Chicago, IL 60657, USA; ^2^Department of Internal Medicine, Wayne State University/Detroit Medical Center, Detroit, Michigan, USA

## Abstract

Aortic dissection is the most devastating sequelae of aortopathy other than aortic rupture. However, aortic dissection can be asymptomatic in the acute phase with delayed symptomatic presentation or incidental diagnosis upon chest imaging. We report a case of a 63-year-old male who was diagnosed with pericardial effusion upon preoperative workup for elective cholecystectomy. Further investigations confirmed hemorrhagic pericardial effusion secondary to a chronic dissecting ascending aortic aneurysm. The patient condition was successfully managed with open surgical repair with an uneventful postoperative course. This case demonstrates an extremely rare presentation of incidental hemorrhagic pericardial effusion caused by a chronic dissecting ascending aortic aneurysm.

## 1. Case Report

**Figure 1:**
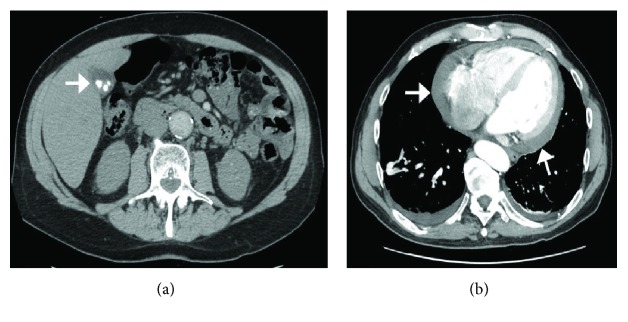
CT scan of the abdomen. (a) Showing gallstones (arrow) with questionable gallbladder wall thickening or pericholecystic fluid. (b) Moderate amount of fluid in the pericardial space appears to be of increased density compared to simple fluid, suggestive of hemorrhage (arrow).

**Figure 2:**
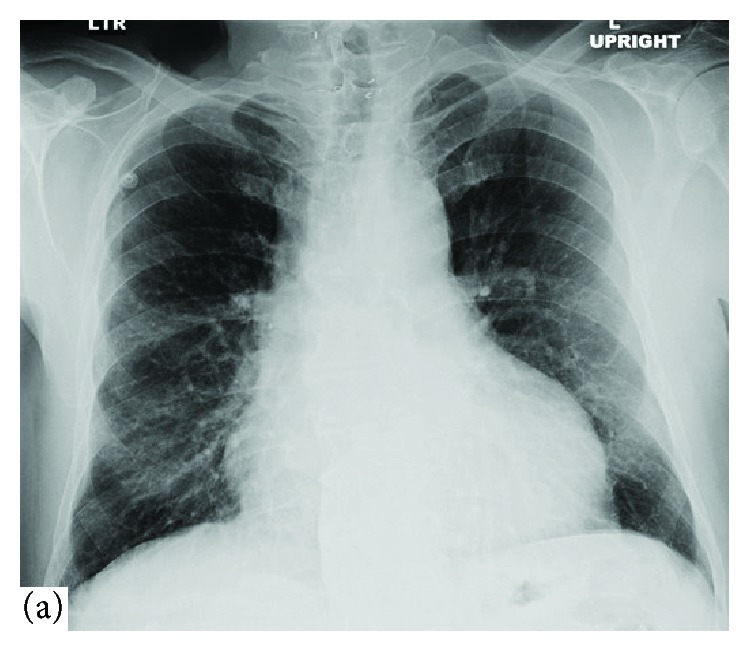
CXR of the lungs (posteroanterior view) on admission shows a mildly enlarged cardiomediastinal silhouette that resembles a bottle.

**Figure 3:**
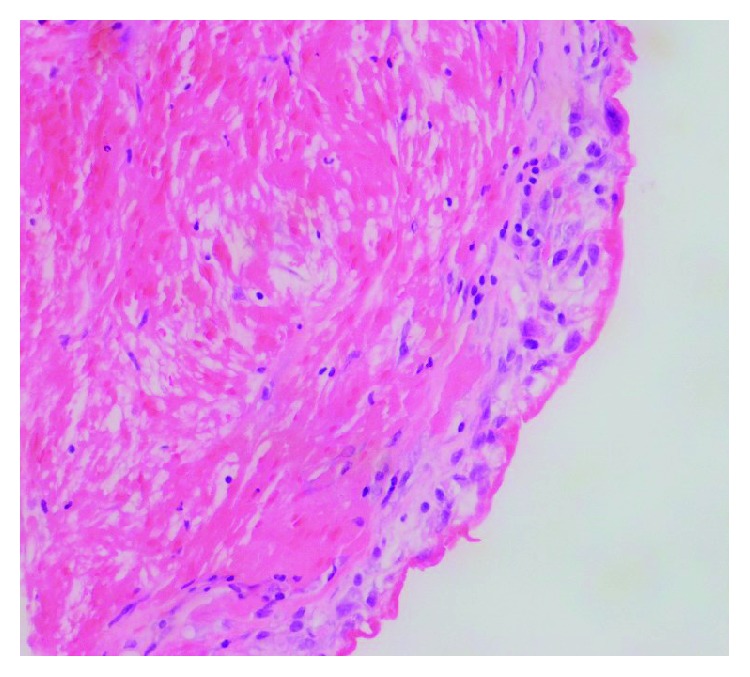
H&E stain of biopsy of pericardial tissue showing thickened pericardial tissue with acute and chronic inflammation and reactive mesothelial hyperplasia.

**Figure 4:**
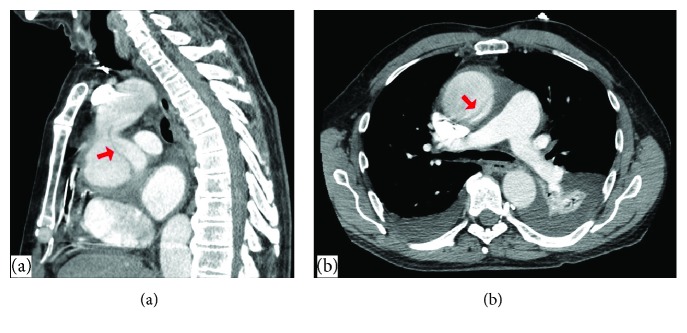
CT scan of the abdomen showing acute ascending thoracic aortic aneurysm measuring up to 7.5 cm with a dissection of the proximal ascending thoracic aorta originates approximately 1.3 cm above the aortic root. (a) Sagittal view of dissection site and (b) coronal view of dissection site. The area of dissection highlighted with an arrow.

A 63-year-old male with a history of hypertension, 20 pack-years of smoking, thyroid cancer in remission with partial thyroidectomy, and chronic obstructive lung disease (COPD) presented to the emergency department (ED) with acute abdominal pain. Abdominal ultrasound showed gallstones with no evidence of cholecystitis or biliary obstruction. The patient was diagnosed with biliary colic and discharged home with a plan to perform an elective cholecystectomy. Computed tomography (CT) scan of the abdomen was performed as an outpatient as part of the preoperative workup which showed evidence of gallstones, small bilateral pleural effusions, and moderate pericardial effusion with high density suggestive of hemorrhage (Figure 1). Consequently, the patient was referred to the emergency department for further workup. Upon arrival, the patient was asymptomatic, his vital signs were normal, and his physical examination was unremarkable. An electrocardiogram (EKG) revealed a nonspecific interventricular conduction delay. Chest x-ray showed a mildly enlarged cardiomediastinal silhouette with prominent perihilar opacities suggestive of a prominent vasculature (Figure 2). Complete blood count and basic metabolic profile were within normal limits. Further laboratory workup showed normal erythrocyte sedimentation rate, rheumatoid factor, complement level, and procalcitonin with negative serology for antinuclear antibodies and double-stranded DNA antibodies. A transthoracic echocardiogram (TTE) showed a large pericardial effusion without signs of tamponade, moderate aortic regurgitation, and normal left ventricular ejection fraction and size. Given the large volume of pericardial effusion, a pericardial window was attempted. A total of one liter of hemorrhagic fluid was drained. Pericardial tissue biopsy showed acute and chronic inflammatory cells with thickened pericardium, and no malignant cells were detected (Figure 3). Tuberculosis quantiferon assay, acid-fast bacilli staining, and fungal and bacterial cultures of the pericardial tissue were negative. The source of pericardial effusion remained elusive. Given the hemorrhagic nature of the pericardial effusion in the absence of recent use of anticoagulation and the negative workup for infectious or autoimmune etiology, a CT scan of the chest with contrast was obtained to evaluate for possible malignancy. CT showed an ascending aortic aneurysm measuring up to 7.5 cm with chronic dissection with no involvement of the coronaries or the great vessels (Figure 4). The patient underwent successful open surgical repair of his chronic Stanford type A aortic dissection with an uneventful postoperative course.

## 2. Discussion

Acute Stanford type A aortic dissection (AD) is a life-threatening condition that serves as an impetus to immediate surgical repair aiming to prevent dissection-associated complications such as acute aortic regurgitation, aortic rupture, myocardial or cerebral ischemia, and pericardial tamponade [1]. However, newly diagnosed chronic type A (CTAD) dissections of a native aorta represent a different subpopulation of patients with atypical or absent symptoms at the time of dissection onset precluding the early diagnosis [2–4]. Patients with de novo chronic AD are usually asymptomatic at the time of diagnosis (like our patient) [2]. Thus, newly diagnosed CTAD is usually discovered incidentally upon chest imaging as a prominent aortic knob or mediastinal widening [2]. Therefore, the accurate timing of dissection is often unfeasible [2]. Careful history is needed to identify previous acute chest pain events (heralding dissection onset). Furthermore, the patient may endorse symptoms related to the enlarging dissection (e.g., hoarseness and persisting or recurrent chest pain), malperfusion (e.g., vision changes, extremity pain, and syncope), or valvular disease (e.g., dyspnea, orthopnea, and lower limb swelling) [1, 2, 5]. Amid any suspicion, CTAD has to be confirmed by further specific imaging modalities including transesophageal echocardiogram (TEE), Computed Tomographic Angiography (CTA), or Magnetic Resonance Angiography (MRA) [1, 2, 5]. Features like thick, immobile intimal flap, aneurysmal dilatation of the thoracic aorta (as in our patient), or the presence of thrombus in the false lumen are suggestive of dissection chronicity [2]. Signs of contained rupture such as pleural effusion or mediastinal hematoma can also be seen in symptomatic patients [2]. Pericardial effusion is a common complication of acute type A AD and usually occurs through two separate mechanisms [5]. The first mechanism is the transudation of fluid across the thin wall of the false lumen into the pericardial cavity which is the most common mechanisms [5]. However, the dissected aorta can rupture directly into the pericardial space with subsequent tamponade [5]. Cardiac tamponade is diagnosed in 8% to 10% of patients presenting with acute type A aortic dissection and is a predictor of poor prognosis, as well as the primary cause of mortality in this subgroup of patients mandating urgent aortic repair [5]. In our case, CTAD was asymptomatic and initially was not clinically suspected; thus, pericardial puncture was carried out for the evaluation of the pericardial effusion. Our patient was hemodynamically stable with no contraindications for contrast administration. Given the negative pericardial fluid analysis, the 20 pack-years of smoking, and the thyroid cancer history, hemorrhagic pericardial effusion secondary to metastatic cancer was suspected. Thus, a CT scan of the chest with contrast was obtained and aortic dissection was incidentally discovered.

The first clue for CTAD diagnosis was the detection of high-density pericardial effusion note on abdominal CT scan before elective cholecystectomy. Given the lake of symptoms, transudation of fluid across the wall of the false lumen into the pericardial cavity is the most plausible mechanisms. Also, hemopericardium in our case could be a sign of silent contained rupture.

All patients with aortic dissections will need immediate surgical evaluation independent of location [5]. Patients with type B dissections are typically treated conservatively [6]. On the other hand, those with type A dissection (like in our case) needs immediate surgical intervention. Acute medical management as a bridge to possible surgery consists of initial pain control and anti-impulsive therapy (i.e., heart rate and blood pressure control) to alleviate the shearing forces against the aortic wall and minimize the propagation of the dissection. The systolic blood pressure goal is less than 120 mm Hg. It is imperative that chronotropic medications be used first as vasodilators pose the risk of reflex tachycardia and worsening of the dissection [6].

Our patient had an aorta diameter of 7.5 cm; furthermore, his dissection was complicated by hemorrhagic pericardial effusion and moderate aortic regurgitation. Thus, the open surgical repair was the chosen approach. According to a recent review of a 696 type A patients, following open repair, patients with CTAD had lower in-hospital mortality and longer five- and ten-year survival compared to the acute type A dissection patients [7]. Our patient had successful CTAD repair with an uneventful postoperative course.

## 3. Conclusion

Chronic type A aortic dissection is a challenging diagnosis with a wide range of clinical presentations including incidental hemorrhagic pericardial effusion. Pericardial puncture in pericarial effusion complicating type A dissection can result in the propagation of the rupture. Accordingly, obtaining a CT scan of the chest prior to any diagnostic pericardial puncture is highly recommended. Surgical repair is the treatment of choice for complicated chronic type A aortic dissection.
